# Stuporous State in a Dog Following Ingestion of Chewing Gum Containing Xylitol Without Documented Hypoglycemia

**DOI:** 10.1002/ccr3.71406

**Published:** 2025-11-03

**Authors:** Laurence M. Saint‐Pierre, Kate Hopper, Jessica M. Jones, Avalene Wan Khoon Tan

**Affiliations:** ^1^ Department of Veterinary Surgical and Radiological Sciences School of Veterinary Medicine University of California Davis California USA; ^2^ Small Animal Specialist Hospital Sydney New South Wales Australia

**Keywords:** hypoglycemic encephalopathy, neuroglycopenia, neurointoxication, reversible encephalopathy, supportive care

## Abstract

An adult dog presented for evaluation of a stuporous state following the ingestion of more than 50 xylitol‐containing chewing gums (minimum xylitol dose ingested > 3 mg/kg). On admission, the patient was hyperglycemic (9.0 mmol/L [163 mg/dL]) with moderately increased liver parameters and a normal plasma ammonia concentration. Non‐ambulatory status, absent menace response bilaterally, and absent gag reflex were noted. Hypoglycemia was never documented throughout hospitalization, although dextrose supplementation was provided when the blood glucose concentration was the lowest (4.2 mmol/L [75 mg/dL]). Within 15 h of supportive care, the dog's neurologic status acutely improved to bright and alert. The patient was discharged home 2 days later. This case describes a dog with severe neurological signs after ingestion of a large amount of gum containing a low dose of xylitol in the absence of documented hypoglycemia and represents the first report of a prolonged yet reversible alteration in mental status under these circumstances, suggesting that xylitol may be neurotoxic even at low doses.

**Abbreviations**
IV: intravenous
LRS: Lactated Ringer's solution
MRI: magnetic resonance imaging
PT: prothrombin time
PTT: partial thromboplastin time
RI: reference interval

**Summary**

- This case highlights that dogs may develop severe neurologic signs after ingesting large amounts of gum containing low xylitol concentrations, even without hypoglycemia.
- Clinicians should recognize potential toxicity, consider non‐hypoglycemic mechanisms, and provide supportive care with hepatic and coagulation monitoring to guide treatment and improve outcomes.

## 1 Introduction

Xylitol is a natural sugar alcohol used as a low‐calorie replacement for sucrose in sugar‐free products such as chewing gum, vitamin supplements, and baked goods. The interest in xylitol as a nutritive sweetener in humans relies on its minimal effects on blood glucose and insulin levels compared to sucrose. These characteristics make xylitol use appealing for patients with diabetes. Beyond its utility in the food industry, the oral antimicrobial properties of xylitol are also well recognized. Xylitol, therefore, remains an essential component of dental care products in human medicine [1, 2].

While it is relatively safe in humans, with reported adverse effects relating to gastrointestinal bloating and diarrhea, it can be life‐threatening when consumed by dogs [3]. Contrary to its minimal impact on insulin in humans, xylitol ingestion in dogs causes insulin release greater than an equivalent dose of glucose, causing severe hypoglycemia [4]. In some cases, ingestion can result in idiosyncratic hepatic failure, although the underlying pathophysiology of hepatic injury with xylitol is not entirely understood [3].

Hypoglycemia is the most well‐recognized consequence of xylitol ingestion in dogs. Neurological signs such as lethargy, ataxia, and seizures are well described and have been attributed to neuroglycopenia at doses exceeding 30 mg/kg [5, 6]. In the veterinary literature, two reports briefly describe dogs presented with lethargy following xylitol intoxication in the absence of hypoglycemia [3, 5]. In one report, patients had concurrent gastrointestinal or cardiovascular signs, which were believed to be the reason for the observed lethargy by the authors. Another report briefly mentions that blood glucose concentrations measured after a seizure episode were within normal limits. This raises the question of underlying mechanisms leading to neuronal dysfunction that could be unrelated to the blood glucose concentration. These earlier reports do not provide a detailed description of the clinical course of these cases. To better characterize this atypical presentation, we provide a detailed case description of a dog that experienced severe neurologic signs after ingesting large amounts of gum containing low doses of xylitol without documented hypoglycemia.

## 2 Case History/Examination

A 9‐year‐old, 33.5 kg, male neutered Golden Retriever presented to the University of California Davis William R. Pritchard Veterinary Medical Teaching Hospital for vomiting and severe obtundation. The patient had ingested an unknown amount of xylitol‐containing chewing gum (Orbit sugar‐free peppermint chewing gum, Wrigley Company, Chicago, IL) 2–4 h prior. The owner reported that a large box of peppermint gum, containing 12 packs with 14 pieces each, for a total of 168 pieces, had been destroyed. According to the manufacturer each gum piece contained 0.2 mg of xylitol. The owner was unaware of any other potential toxins that the dog could have ingested. On presentation, the patient was laterally recumbent, stuporous, slightly hypothermic (37.2°C [99.1 F]), tachycardic (140 beats per minute) with strong femoral pulses, and hyperemic mucous membranes. The neurologic evaluation showed non‐ambulatory status and intact cranial nerve function, with the exception of an absent menace response and gag reflex.

## 3 Methods

Given the history and clinical signs, the patient was presumed hypoglycemic. A blood sample was collected to measure the glucose concentration (AlphaTRAK, Blood Glucose Monitoring System, Zoetis, Parsippany‐Troy Hills, NJ) and the dog received an intravenous (IV) dextrose (Dextrose 50%, MWI, Boise ID) bolus (7.5 g, 0.2 g/kg) before the results were known. The dog was later found to have been hyperglycemic 9.0 mmol/L (163 mg/dL, Reference interval (RI): 4.2–6.7 mmol/L [75–120 mg/dL]) before dextrose administration. Pertinent findings of the venous blood gas panel (ABL 800 Flex, Radiometer Medical A/S, Copenhagen, Denmark) post dextrose bolus were PvO_2_ 66.4 mmHg (RI: 30–50 mmHg), potassium 2.7 mEq/L (2.7 mmol/L, RI: 3.5–4.8 mEq/L [3.5–4.8 mmol/L]), ionized calcium 1.46 mmol/L (5.85 mg/dL, RI: 1.3–1.46 mmol/L [5.21–5.85 mg/dL]), glucose 18.5 mmol/L (333 mg/dL) and lactate 5.6 mmol/L (RI: < 2 mmol/L). The patient was administered 1 L (29.8 mL/kg IV) of Lactated Ringer's solution (Lactated Ringer's, Baxter, Deerfield, IL) (LRS) as a bolus and kept on LRS at 20 mL/kg/h for an additional 1.5 h for further cardiovascular support. Biochemistry parameters (Cobas c501, Roche Diagnostics, Basel, Switzerland) taken after the initial crystalloid bolus showed a mild increase in bilirubin, moderate increase in alanine aminotransferase (ALT), and aspartate aminotransferase (AST). Plasma ammonia concentration was 19.9 μmol/L (34 μg/dL, RI: < 34.6, μmol/L [< 59 μg/dL]) (Table 1). A CBC taken at that time was normal except for mild bandemia (0.08 × 10^9^/L (85/μL), RI: rare), lymphopenia (0.6 × 10^9^/L, 595/μL, RI: 1.0–4.0 × 10^9^/L [1000–4000/μL]) and slight hemolysis. The dog remained stuporous for the next 14 h. Plasma glucose was measured every 2 h for the first 12 h, then every 4 h for another 12 h, and remained between 4.2 and 12.4 mmol/L (75–223 mg/dL). Dextrose supplementation (5%) was prophylactically started 5 h after admission when the blood glucose was the lowest (4.2 mmol/L [75 mg/dL]) and was continued for a duration of 10 h. No change in mentation was noted after dextrose supplementation.

**TABLE 1:** Chemistry and coagulation parameters at admission and throughout hospitalization in a dog after ingestion of a large amount of xylitol‐containing gums.

Parameters	Admission	15 h post admission	48 h post admission	Reference intervals
Total protein, mg/dL [g/dL]	45.0 [4.5]	52.0 [5.2]	N/A	54.0–69.0 [5.4–6.9]
Albumin, mg/dL [g/dL]	27.0 [2.7]	35.0 [3.5]	N/A	34.0–43.0 [3.4–4.3]
Glucose, mmol/L [mg/dL]	9.0 [163]	12.4 [223]	N/A	4.2–6.7 [75.0–120.0]
BUN, mmol/L [mg/dL]	3.9 [11.0]	4.3 [12.0]	N/A	3.9–11.8 [11.0–33.0]
Cholesterol, mmol/L [mg/dL]	7.1 [276.0]	8.5 [327.0]	N/A	3.6–9.14 [139.0–353.0]
Bilirubin, μmol/L [mg/dL]	5.1 [0.3]	3.4 [0.2]	N/A	< 3.4 [< 0.2]
AST, U/L	854.0	1388.0	N/A	20.0–49.0
ALT, U/L	326.0	547.0	N/A	21.0–72.0
ALP, U/L	50.0	64	N/A	14.0–91.0
GGT, U/L	< 3.0	3.0	N/A	0.0–5.0
Phosphorus, mmol/L [mg/dL]	0.2 [0.7]	0.9 [2.7]	N/A	0.8–1.7 [2.6–5.2]
PT, s	N/A	> 120.0	14.3	7.0–9.3
aPTT, s	N/A	> 140.0	19.6	10.4–12.9
Fibrinogen, g/L [mg/dL]	N/A	< 5.0 [< 50]	5.1 [51]	10.9–3.1 [109.0–311.0]
Ammonia, μmol/L [μg/dL]	19.9 [34.0]	N/A	N/A	< 34.6 [< 59.0]

Abbreviations: ALP, Alkaline phosphatase; ALT, alanine aminotransferase; aPTT, activated partial thromboplastin time; AST, aspartate aminotransferase; GGT, gamma‐glutamyl transferase; N/A, not available; PT, prothrombin time.

The dog was administered crystalloids (Plasma‐Lyte A [Plasma‐Lyte A, Baxter, Deerfield, IL], 1.5 mL/kg/h IV for 53 h) supplemented with potassium phosphate (Potassium phosphate, Hospira Inc., Lake Forest, IL) (40 mEq/L of potassium and 20 mmol/L of phosphorous) and N‐Acetyl cysteine (Acetylcysteine 20%, American Regent, Shirley, NY) (140 mg/kg followed by 70 mg/kg IV every 6 h). Three‐lead continuous ECG showed sinus rhythm with occasional ventricular premature complexes and non‐sustained accelerated idioventricular rhythm. The patient vomited approximately 50 chewing gum pieces 4 h after admission while remaining stuporous. This finding enabled estimation of a minimum xylitol dose of 3 mg/kg.

## 4 Conclusion and Results

Fourteen hours after admission, the patient's mentation acutely improved from stupor to bright, alert, and ambulatory without overt ataxia. At that time, ecchymoses were noted around the IV catheter site and on the flank where the ECG pads were initially placed. A coagulation panel (STA compact max, Stago, Asnières sur Seine, France) 15 h post‐admission showed marked abnormalities (Table 1). A chemistry panel and complete blood count were repeated at that time and showed a moderate increase in ALT and AST (Table 1). The platelet count was 37.0 × 10^9^/L (37,000/μL RI: 150–400 × 10^9^/L [150,000–400,000/μL]) with clumps and rare macroplatelets. The patient was started on vitamin K1 (Vitamin K1, MWI, Boise, ID) (37.5 mg orally every 12 h) and Denamarin (Denamarin, Nutramax Laboratories, Lancaster, SC) (670 mg orally once daily). Coagulation parameters improved on Day 3 of hospitalization (PT 18.5 s RI: 7.0–9.3 s, aPTT 23.2 s RI: 10.4–12.9 s, fibrinogen 3.0 g/L [30 mg/dL] RI: 10.9–31.1 g/L [109–311 mg/dL]). The dog remained bright and alert with a normal appetite and was discharged home on Day 3 with Denamarin (650 mg once daily orally for 7 days) and vitamin K1 (37.5 mg orally twice daily for 21 days). A recheck coagulation panel was performed 6 days after discharge (PT 10.7 s, aPTT 13.2 s, fibrinogen 8.2 g/L [82 mg/dL]), and complete normalization was noted on the last recheck, 24 days later (PT 8.6 s, aPTT 11.6 s, fibrinogen 13.6 g/L [136 mg/dL]).

## 5 Discussion

This report describes the clinical course of a dog who developed a severe stupor after ingesting a large quantity of xylitol‐containing gums without documented hypoglycemia. Albeit severe, the clinical signs resolved after 14 h of supportive care, and the dog was discharged home after 3 days of hospitalization.

In dogs, clinical signs of xylitol intoxication typically develop within 30 to 60 min of ingestion. These signs can be either gastrointestinal (vomiting, diarrhea) and/or neurologic (lethargy, ataxia, weakness, or seizure activity) in nature. The neurologic signs are most commonly secondary to severe hypoglycemia, a phenomenon referred to as neuroglycopenia. The brain heavily depends on glucose as a source of energy and has a limited ability to use other substrates. Therefore, a continuous supply of glucose must be maintained across the blood–brain barrier for adequate cerebral function [7]. Neuroglycopenia is defined as a state in which there is an insufficient glucose supply to the brain. It typically occurs when the blood glucose concentration falls below 36–51 mg/dL (2.0–2.8 mmol/L) in people and 18–34 mg/dL (1.6–1.9 mmol/L) in dogs [8, 9, 10]. Clinical signs described in humans include weakness, drowsiness, confusion, and, in severe cases, coma and death [10]. In general, short hypoglycemic episodes result in reversible and non‐life‐threatening neurological deficits. However, prolonged or severe neuroglycopenia may lead to neuronal death and potentially irreversible neurologic signs, a syndrome called hypoglycemic encephalopathy [11]. The hypoglycemic syndrome in xylitol intoxication results from metabolites stimulating insulin secretion. It was once believed to occur at doses of 0.1 g/kg but was later shown in a case series to develop at a minimum dose of 0.03 g/kg in dogs [5]. Once ingested, xylitol can be rapidly metabolized to D‐xylulose, a key player in insulin secretion [12]. In canine experimental studies, a dose‐dependent insulin release peaks 45 min after xylitol ingestion and is of greater magnitude than observed with an equivalent dose of glucose [4]. This marked insulin secretion leads to the hypoglycemia observed in these patients. However, for neurological signs to be attributed to hypoglycemia, all three criteria of Whipple's triad must be met. These include (1) symptoms of hypoglycemia, (2) documentation of low plasma glucose at the time of clinical signs, and (3) resolution of clinical signs with correction of hypoglycemia [7]. In the present report, the patient exhibited severely altered mental status in the absence of documented hypoglycemia. These abnormalities persisted for 15 h despite the lowest measured blood glucose concentration of 4.1 mmol/L (75 mg/dL). Therefore, according to Whipple's triad and the suspected ingested dose, neuroglycopenia was unlikely to be the cause of the neurologic signs observed in this case.

Hepatic encephalopathy is another mechanism of altered neurologic status in xylitol‐intoxicated dogs [13, 14]. Xylitol intoxication leads to a moderate to marked increase in ALT and AST in dogs, suggesting hepatic damage due to ATP depletion from xylitol metabolism [15]. In some cases, idiosyncratic liver failure is noted and can occur as early as 9 h post‐ingestion. However, signs consistent with hepatic encephalopathy were documented only 24 h after ingestion in one report [14]. Hepatic encephalopathy is described as a complication of acute or chronic liver insufficiency. It is characterized in dogs by obtundation, ataxia, seizures, stupor, and tremors, among other clinical signs [16]. The diagnosis of hepatic encephalopathy secondary to acute liver failure is mainly clinical. It relies on consistent clinical signs, exclusion of other causes of encephalopathy, laboratory findings of acute liver failure, and response to treatment [16]. In this report, although the moderate to marked AST and ALT increase suggested some degree of liver damage, the classic markers of acute hepatic failure were not present on admission. These include profound hypoglycemia and hyperbilirubinemia and indicators of hepatic encephalopathy such as hyperammonemia. Moreover, in reports of dogs with acute hepatic failure from xylitol intoxication, the degree of elevation of bilirubin, ALT, and AST was more profound than in this dog (bilirubin 17.1–85.5 μmol/L [1.0–5.0 mg/dL], ALT 1000 to > 10,000 U/L and AST 3251 to > 25,000 U/L) [14, 17]. The patient presented in a stuporous state within 4 h of xylitol ingestion before liver failure and significant hepatic encephalopathy would occur. Moreover, the stupor resolved without specific treatment for hepatic encephalopathy. Therefore, it is unlikely that this syndrome played a significant role in the neurologic signs observed in this dog.

The dog presented here had abnormal coagulation parameters documented during hospitalization. The cause of these changes is unclear. Coagulation changes are a component of liver failure, which can be a consequence of xylitol ingestion, but as discussed, liver failure was not evident in this case [14, 18]. The coagulation abnormalities appeared to be transient and self‐resolving. The presence of coagulation changes raises the possibility of intracranial bleeding as a cause of the neurologic abnormalities. However, the rapid onset of the mentation changes in this dog and the lack of evidence of bleeding during the initial case management suggest that the coagulation changes developed later in the disease process.

While there is mention of isolated cases of neurologic signs in the absence of hypoglycemia in 2 reports, these cases were not described in detail [3, 5]. In the veterinary literature, mild neurologic signs such as lethargy in the absence of hypoglycemia were briefly mentioned in 7/192 dogs intoxicated with xylitol [5]. However, the authors attributed these mentation changes to concurrent gastrointestinal or cardiovascular abnormalities. In a review on xylitol intoxication, the authors stated, “dogs that were presented either nonresponsive or with seizure activity were often hypoglycemic, although in some cases, blood glucose levels performed postictally showed levels within the normal limits” [3]. These isolated cases were not extensively described, especially regarding the progression of the neurologic signs over time. Our detailed report raises the question of another mechanism leading to neuronal dysfunction during xylitol intoxication in the absence of documented hypoglycemia. We propose two potential mechanisms to explain these findings.

In humans, xylitol is considered safe, and the side effects reported are related to the gastrointestinal system. However, a few case reports describe the development of more serious adverse effects when xylitol was used as a form of parenteral nutrition. These include renal and liver dysfunction, cerebral disturbances, and oxalate crystal deposition [19, 20, 21]. In these patients, varying degrees of neurologic signs were noted. These include nausea, drowsiness, irrational behavior, aphasia/dysphasia, or even progressive obtundation leading to coma and death. The clinical signs worsened or resolved spontaneously within 48–72 h after cessation of the infusion in some patients [19]. Post‐mortem histologic evaluation was variable. While one patient had no histologic abnormalities, calcium oxalate deposits in the walls of small cerebral arterioles were noted in two patients receiving xylitol‐containing parenteral nutrition [19, 21]. At this time, the mechanism of cerebral dysfunction is not entirely understood, and the significance of the calcium oxalate deposits remains unclear. Nevertheless, these reports highlight the possibility of a direct effect of xylitol on the central nervous system that may have occurred in the dog presented in this report. Further research is needed to fully understand the underlying mechanism of such neurologic dysfunction.

A second possible mechanism for the observed clinical signs involves the development of hypoglycemic encephalopathy. Although the patient was normoglycemic on admission, we cannot exclude that a severe transient hypoglycemic episode occurred at home, causing persistent neurologic abnormalities. Experimental studies in dogs with xylitol intoxication showed severe hypoglycemia (1.44–2.03 mmol/L [26–36.5 mg/dL]) 1 h after ingestion and blood glucose normalization 120–150 min post ingestion without dextrose supplementation [4, 15]. However, in these reports, the clinical signs are not precisely described throughout the experiment. One report describes that “most dogs became inactive and depressed after xylitol dosing,” although there is no mention of the dogs' neurologic status after normalization of the blood glucose [15]. Interestingly, human patients can remain comatose or stuporous after a severe yet transient episode of hypoglycemia even after normalization of their blood glucose [22]. In this syndrome called hypoglycemic encephalopathy, patients who show improvement in their mental status generally experience a shorter duration of hypoglycemia (1.6 vs. 7.8 h) [22]. Transient or irreversible cortical lesions are documented on magnetic resonance imaging (MRI), such as necrosis of the gray matter. This phenomenon has not been clearly documented in dogs. One case report showed MRI lesions consistent with this syndrome in a dog with status epilepticus [23]. Another report describes necropsy findings in a dog with insulinoma, in which hypoglycemic encephalopathy was suspected [24]. In both reports, the dogs died due to the consequences of this syndrome. It remains unclear if such a phenomenon occurred in the dog presented in this report, as an MRI was not performed, especially since the ingested xylitol dose was low and not expected to cause hypoglycemia. Given that the clinical signs could resolve with supportive care, the clinician should be aware of such a mechanism when dealing with similar cases.

Induction of emesis is generally considered futile in xylitol toxicosis due to the rapid gastrointestinal absorption within minutes. Xylitol carriers were vomited up after more than 5 h post‐ingestion in this case. The persistence of ingested material beyond the time frame for normal gastric emptying is likely due to xylitol‐induced gastric ileus. Numerous reports in human medicine discuss the effect of xylitol, either as a part of a “solid‐food complex meal” or as a liquid, on gastric emptying rates [25]. Xylitol induces a dose‐dependent deceleration of gastric emptying in humans by stimulating the release of cholecystokinin and active‐glucagon‐like‐peptide 1 [25]. Gastric decontamination via emesis or gastric lavage, if safe to do so, may be beneficial if solid xylitol‐containing products such as gum pieces are ingested, especially if severe and direct neurologic effects are suspected.

This report has several limitations, the most significant being the uncertainty about which substance in the gum caused the neurological signs. The gum primarily contained sorbitol, gum base, a small amount of aspartame, and xylitol. Neurological toxicity has not been reported with sorbitol in dogs, and although aspartame has been associated with neurological effects in mice at doses of 250 mg/kg, such effects have not been documented in dogs [26]. Additionally, while a large quantity of gums was vomited, the patient could have concurrently consumed other toxicants as the ingestion event was not witnessed. However, this was considered unlikely after thorough questioning of the owner.

We describe the clinical course of severe yet reversible neurological abnormalities following consumption of a large quantity of xylitol‐containing gums without documented hypoglycemia. It highlights the importance of measuring blood glucose in these patients and describes mechanisms leading to severe yet reversible neurological dysfunction despite normal blood glucose concentrations, even at low xylitol exposure. It also raises the consideration of gastric decontamination when clinical signs are severe and when there is evidence of persistent solid xylitol‐containing material in the stomach. Monitoring liver function and providing appropriate supportive care is another crucial aspect of the care of these cases, as has been described previously.

## Author Contributions

**Laurence M. Saint‐Pierre:** conceptualization, data curation, investigation, methodology, resources, writing – original draft, writing – review and editing. **Avalene Wan Khoon Tan:** validation, writing – original draft, writing – review and editing. **Jessica M. Jones:** validation, writing – original draft, writing – review and editing. **Kate Hopper:** supervision, validation, writing – original draft, writing – review and editing.

## Consent

Written consent was obtained from the dog's owner.

## Conflicts of Interest

The authors declare no conflicts of interest.

## Data Availability

The data that support the findings of this study are available from the corresponding author upon reasonable request.
